# *In Vivo* Anti-*Candida* Activity of Phenolic Extracts and Compounds: Future Perspectives Focusing on Effective Clinical Interventions

**DOI:** 10.1155/2015/247382

**Published:** 2015-08-24

**Authors:** Natália Martins, Lillian Barros, Mariana Henriques, Sónia Silva, Isabel C. F. R. Ferreira

**Affiliations:** ^1^Mountain Research Centre (CIMO), ESA, Polytechnic Institute of Bragança, Campus de Santa Apolónia 1172, 5301-855 Bragança, Portugal; ^2^Centre of Biological Engineering (CEB), Laboratório de Investigação em Biofilmes Rosário Oliveira (LIBRO), University of Minho, Campus de Gualtar, 4710-057 Braga, Portugal

## Abstract

*Candida* species have increasingly deserved a special attention among the medical community. In spite of the presence of *Candida* species as a human commensal, alarming rates of local and systemic infections have been observed, varying from moderate to severe impact. Currently available antifungal drugs have progressively lost their effectiveness, pointing urgently the problem of the microorganisms with acquired-resistance. Natural matrices are secularly used for numerous purposes, being inclusive and highly effective as antimicrobials. Increasing evidence gives a particular emphasis to the contribution of phenolic extracts and related individual compounds. *In vitro* studies clearly confirm their prominent effects, but the confirmation through *in vivo* studies, including the involved mechanisms of action, is not so much deepened. Therefore, the present report aims to provide extensive knowledge about all these aspects, highlighting the most efficient phytochemical formulations, including therapeutic doses. Further studies need to be incited to deepen knowledge on this area, namely, focused on clinical trials to provide safer and more effective antimicrobials than the current ones.

## 1. Introduction

Over the last years, plant-derived extracts and related phytochemicals have gained a particular attention by scientific researchers, due to their healing effects [1–3]. Studies involving the elucidation of their mechanisms of action, including pharmacokinetics and pharmacodynamics, have been also performed [4–8]. In the last two decades, among its phytotherapeutic properties, the antimicrobial potential, namely, anti-*Candida* effects, has deserved a particular attention. In fact,* Candida* species have been implicated in an onset of mild and severe clinical conditions, although it was considered a commensal microorganism of healthy individuals [9].

In the majority of cases, patients have no infection signals and symptoms manifestation; thus, upon a clinical diagnosis, a severe colonization and related infection are installed [9]. Antifungals and even other antimicrobial drugs are, therefore, extensively prescribed; but the present condition is much more complex than simple* Candida* species overgrowth [9]. Other triggering factors are also involved, which are neither considered nor regulated by the antimicrobials. Obviously, recurrent infections will be installed, leading to the appearance of microorganisms highly resistant to conventional antifungal drugs [10–12].

Plants and all living organisms produce countless substances aiming at their survival, defence, nutrition, and even growth [13–15]. Regarding their antimicrobial properties, namely, antifungals, some of them have already been described. Phenolic compounds, namely, phenols, flavonoids, coumarins, quinones, saponins, and xanthones, are the most abundant, besides alkaloids, lectins, polypeptides, terpenoids, and essential oils [15–18].* In vitro* studies are crucial to screen their effects [19], security, efficiency, and other biochemical parameters, but plant-derived bioactive molecules possess several modes of action, establishing synergic, antagonist, and polyvalent relationships with other compounds, besides suffering chemical changes due to organic metabolism, including the effects on gut microbiota [5, 20, 21]. Those features could not be assessed through* in vitro* and, therefore,* in vivo *studies, and mostly clinical trials shall be also considered.

In this sense, the present report intends to provide extensive knowledge on* in vivo* anti-*Candida* potential of phenolic extracts and compounds. Furthermore, the* in vivo* mechanisms of action of the previous will be presented, as well as potential clinical applications (i.e., galenical formulations, among others).

## 2. *Candida* Species: Clinical Impact

### 2.1. Epidemiological Features

Microorganisms are ubiquitous and, in adequate concentrations, are crucial to the organic homeostasis. To ensure this equilibrium, friendly microbiota, gastrointestinal pH, immune system, and organic metabolism play a role of the utmost importance [22, 23]. Nevertheless, daily routine, environmental factors, and people lifestyles, among other factors, act as triggers to unbalance. Additionally, higher levels of stress, the overuse of antibiotics, antacids and proton-pump inhibitors, birth control pills or steroids, the exposure to environmental chemicals, and wrong food choices (namely, diets with too many sugars, alcohol, and fermented products) are encountered between the most common causes of dysbiosis. Much more than a “*dys*” (which means not) and “*symbiosis*” (which means living together in mutual harmony), dysbiosis reflects the relationship between microbes and the host [22, 23]. In contrast with some high-virulence microorganisms that cause immediate reactions, low-virulence microorganisms are insidious.* Candida* species, considered a commensal microorganism, comprises an excellent example [22–24]. Among them,* C. albicans* is encountered as the most frequent, being responsible for approximately 50–90% of cases of human infection (candidiasis) [9]. But presently, other non-*albicans Candida* species have also been involved, mainly* C. tropicalis*,* C. glabrata*,* C. dubliniensis,* and* C. parapsilosis*. Besides their associated virulence features, they are also able to form biofilms with other species, which not only makes their eradication difficult but also improves their prevalence and related resistance to antimicrobials [9, 25]. Thus, and despite providing several benefits to the host, they possess a higher adaptability and numerous strategies to survive, which not only favors their overgrowth but also changes their susceptibility profiles. The human organism is colonized by these yeast species at, or near, birth, mainly through a physical contact with the vaginal flora [25–27]. Under normal physiological conditions,* Candida* species are present in small amounts, but some individual characteristics determine the probability of* Candida* overgrowth and, consequently, establishment of infection. For example, women are most susceptible, as hormonal variations act as triggering factors, but men could also be affected [9, 28].

Previous to the synthesis of chemical drugs, namely, antibiotics and other antimicrobials, microorganisms naturally produce natural biocides towards each other, but also establishing symbiotic relationships [22, 29, 30]. Nevertheless, due to the disseminated adhesion to the use and overuse of antibiotics, not only in humans but also in animals, numerous microorganisms with undergoing mutations have appeared, and linked with this, the rates of chronic and degenerative problems soared in an exponential manner.

### 2.2. Drug Resistance

A large amount of antimicrobial drugs, such as antibiotics and antifungal agents, is currently available to fight against a wide variety of infectious syndromes [31, 32]. Once these drugs act in a nonspecific way, they kill not only harmful but also helpful microorganisms, including healthy microbiota, apart from interfering with several metabolic pathways [22, 29, 30]. As previously mentioned, opportunistic microorganisms may be present in small amounts in healthy organisms, without causing any clinical disturbance or infectious symptoms. But, unfortunately, in the last decades, in the face of a minimal symptom of infection, antimicrobial drugs are widely prescribed, mostly without determining the causative agent. The fact that unbalanced diets and nutrient deficiencies are important secondary causes of infection is completely ignored [22, 30]. In fact, several nutrients are crucial to maintain a healthy immune system [22, 23, 32]. So, it is easy to understand that, in the face of nutritional deficits, the defense system cannot work properly, and so the probability of occurrence of opportunistic infections and other organic disorders is significantly improved.

In the face of the higher rates of microorganisms with acquired drug resistance, among which are* Candida* species-related infections, scientific advances regarding new synthetic drugs, more specific and effective than the previous, have been supported by pharmaceutical industries. The current ones are ineffective not only at higher doses but also in drug combination [10, 33–35]. The most prominent situation is the acquired-resistance to azoles, mainly by enhancement of its efflux through upregulation of multidrug transporter genes on yeast cells, besides changes in the ergosterol biosynthesis and occurrence of mutations. The new synthetic drugs seem to be effective against the majority of insidious- and resistant-*Candida* species [35–40], but the problem of their side effects and related toxicity still remains.

### 2.3. Upcoming Approaches

Plants comprise an extremely rich pool of bioactive constituents [41–45]; most of them are secondary metabolites (final or intermediate products) [14, 45–47]. Among them, phenolic compounds have gained a special attention in the last years. In fact, numerous studies have pointed their prominent antifungal potential [16, 45, 46, 48–50].

The use of botanical preparations is an ancient practice, but without a solid scientific basis, it is poorly accepted by the medical community. Numerous underlying interests have blocked its worldwide recognition, in spite of its evident effectiveness and absence of side effects and toxicity, when properly used. It is a fact that several studies have reported problematic herbal-drug interactions [51–53], but it is very important to highlight that the majority of synthetic chemicals are derived from plants, that they act on symptoms and not in the predisposing cause(s), that they only possess one or another therapeutic indication, and that the magnitude of side effects and toxicity is directly related with the prescribed dosages [22, 32]. On the other hand, natural matrices, besides providing a wide variety of phytotherapeutic properties, improve and even contribute to a proper nutritional balance [22–24, 54]. Furthermore, these bioactive molecules show a higher spectrum of clinical applications, due to the occurrence of synergic, antagonistic, and polyvalence reactions; their side effects and related toxicity disappeared and/or are neutralized, as well as being able to favor the organic homeostasis, providing not only immunomodulatory and healing properties, but also other health benefits [22, 23, 55]. Thus, while botanical preparations act in a holistic manner, the chemical drugs mainly block one or more metabolic pathways improving the organic unbalance [22–24, 32]. Among the intensive research on this area,* in vitro* studies clearly assume the leadership, while* in vivo* studies remain secondary. Notwithstanding,* in vitro* studies have clear limitations, Notwithstanding,* in vitro* studies have clear limitations; so, apart from the discovery of potentially effective alternatives, is also important to analyze its feasibility and to access the involved modes of action, through* in vivo* studies.

## 3. Clinical Importance of* In Vivo* Studies

### 3.1. Historical Perspective

The diagnosis of an infection comprises several levels of complexity, and currently the idea that the presence of some microorganisms is inoffensive was changed completely [56, 57]. In particular, opportunistic microorganisms, including* Candida* species, can cause from a simple catheter-related fungemia or peritonitis to severe localized infections, or even extensive hematogenous dissemination [9, 57, 58]. Immunocompromised patients, transplanted or submitted to broad surgeries, individuals with neoplastic diseases, and children and elderly people are considered as a higher risk population, being much more vulnerable than other common citizens [9, 58]. In this sense, and considering that in some cases neither signals nor symptoms are observed, it is crucial to establish a correct diagnosis, which implies not only the use of representative isolated clinical material, but also the isolation and identification of the involved microorganism, towards a correct clinical intervention [56, 57].

In the first stages of the clinical investigation,* in vitro* techniques (such as microscopic, serologic, antigenic, amplification, and susceptibility tests) are extremely useful, but it is necessary to highlight that they do not consider numerous variables, namely, the individual idiosyncrasies and organic metabolism. Thus, and regarding the latest advances related with the opportunistic fungal infections, numerous* in vitro* studies have been carried out, including the screening of some natural extracts (e.g., rich in phenolic compounds) with anti-*Candida* potential [19], but also evaluating their mechanisms of actions and other laboratorial parameters (i.e., proteomic, genetic, molecular studies, etc.) [5, 6, 50, 59, 60]. Notwithstanding,* in vivo* studies still remain scarce.

### 3.2. *In Vivo* Antifungal Potential of Phenolic Extracts

Table 1 shows the phenolic extracts with reported* in vivo* activity against* Candida* species.* Combretaceae* followed by* Acanthaceae* are the most studied plant families. Considering the obtained results, leaves, followed by the seeds and fruits, and then flowers appear as the most enriched-plant parts. In fact, leaves and seeds/fruits show higher levels of phenolic compounds, but their concentrations depend greatly on the growth, harvesting, and storage conditions [61–64]. Additionally, the extractability of phenolic compounds depends on the type of solvent used [19]. Methanol, followed by acetone, water, ethanol, petroleum ether, methanol-dichloromethane, ethyl acetate, and n-butanol encounter between the most frequently used extraction solvents. In fact, these solvents were also the most commonly used in the* in vitro* studies dealing with anti-*Candida* potential of phenolic extracts [19].

The neutrophils adhesion, locomotion, and chemotaxis tests, as well as the assessment of their phagocytic activity to kill* C. albicans,* have been some of the parameters used to evaluate the activity of phenolic extracts against* Candida* species [65, 66]. The obtained results allow not only the determination of the abundance of neutrophils in blood samples, in terms of number of total leukocyte cells (TLC) and differential leukocyte cells (DLC), but also the analysis of the efficiency/capacity to kill* C. albicans*. Haemagglutinating antibody (HA) titre and delayed-type hypersensitivity (DTH) response tests have been also carried out [67]. The obtained results from these tests can be considered direct indicators of the status of the immune system and also allow the determination of the immunomodulatory potential of the studied substances. The humoral immune response presupposes the existence of phagocytic activity but also includes other immune-related organic reactions, such as induction of synthesis, reproduction, and differentiation of defense cells. In this sense, the previous assays give specific information related with the activity and efficiency of organic-defense cells to ingest, to remove, and to destroy not only microorganisms, but also malignant cells, inorganic particles, and altered tissues.

Evaluation of the wound healing potential, for example, through measuring the tensile strength, exudate, lesion size, crust formation, and also histological and histopathological examinations, has been applied by some authors [68–75]. In fact, the colonization of wounds by microorganisms is very common; thus, it is extremely useful to discover plant extracts with pronounced abilities to improve, for example, wound closure and crust formation. Among the wide variety of plant's secondary metabolites, flavonoids, but specifically tannins, have marked astringent, antioxidant, and also antimicrobial potential and, therefore, a direct influence in wound contraction and healing potential [69, 73].

The evaluation of the efficiency/efficacy of plant extracts in induced systemic and local infection models is a very interesting approach, once it mimics the real conditions of infected organisms and, at the same time, the achievement of the direct effect of the tested substances. Vijayarathna et al. [76], Jothy et al. [77], Sahgal et al. [75], and Dzoyem et al. [78] evaluated the effects of plant extracts in systemic infections, while Araújo et al. [70] and Simonetti et al. [79] in induced vaginal infection, Abaineh and Sintayehu [80] in local mastitis, and, lastly, Chelli-Chentouf et al. [81] in oral health and improvement decays in children.

Although the aim of the present report is to highlight the* in vivo* anti-*Candida* potential of phenolic extracts and compounds, it should be noticed that natural matrices possess much more than a single bioactivity and that the sum of several bioactivities might result in a different final clinical application. Furthermore, a unique bioactive potential (conferred by a specific or several chemical compounds) is able to improve considerably other bioactive effects. For example, several reports have shown that plant extracts with a significant antioxidant potential also evidence considerable anti-inflammatory and antimicrobial effects [42, 82–85]. Besides, plant extracts with higher antifungal potential normally exert significant immunomodulatory and also antiseptic effects. Thus, and in the same line with the results of several authors, plant extracts that present a considerable healing potential have also a great antioxidant, antiseptic, and antimicrobial activities. Phenolic compounds are the most representative chemical compounds with the above-described bioactive properties, but due to the slight number of* in vivo* studies reporting their anti-*Candida* potential, few conclusions can be stated. Beyond that,* C. albicans* is the main focus of the present studies, but other non-*albicans Candida* species should be also considered once present in different infections.

### 3.3. *In Vivo* Antifungal Potential of Individual Phenolic Compounds

**Figure 1:**
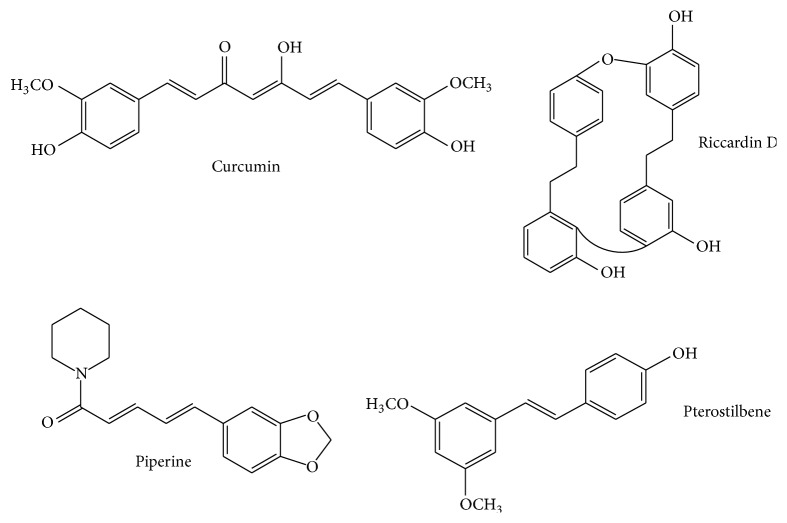
Chemical structures of the tested bioactive molecules with* in vivo* anti-*Candida* potential.

Table 2 shows the* in vivo* antifungal potential of phenolic compounds against* Candida* species. Stilbenes, namely, pterostilbenes and riccardin D, are the most studied phenolic compounds with anti-*Candida* potential, followed by curcuminoids (curcumin and piperine). Its respective chemical structures are shown in Figure 1. Among the tested stilbenes, pterostilbenes evidenced a higher anti-*Candida* biofilm activity than the riccardin. Li et al. [86] observed a total inhibition of the biofilm formation by using concentrations of 32 *μ*g/mL and 64 *μ*g/mL of pterostilbenes; at 16 *μ*g/mL,* C. albicans* biofilms were defective and only sparse cells were founded. In contrast, the same authors [87], evaluating the effect of riccardin D on anti-*Candida* biofilm formation, did not observe a complete growth inhibition by using a concentration of 64 *μ*g/mL.

Concerning the evaluation of the antifungal potential of curcuminoids, Sharma et al. [88] showed that the use of curcumin alone provides an insignificant effect, due to its poor bioavailability, but when administered in association with piperine, an inhibitor of the hepatic and intestinal glucuronidation, its efficacy, and related antifungal efficiency was completely changed. For example, the antifungal potential of associated substances, curcumin (100 mg/kg) in combination with piperine (20 mg/kg), presents a similar potential to the current antifungal drug fluconazole (50 mg/kg).

Nevertheless, considering the scarce number of studied phenolic compounds, no solid conclusions can be proposed. Additionally, commercial standards are encountered as the most studied, alternative to the plant-derived phenolic compounds.* Candida albicans* continues to be the most focused* Candida* species, but concerning the real clinical conditions, for example, catheter infections and systemic infection models, other non-*albicans Candida *species should be also considered. Rats, particularly albino and immunocompromised, have been the mostly used animal models due to not only their higher susceptibility to infections, but also weak efficiency of their immune system and polymedication.* Per se*, the multidrug administration is an isolated triggering factor that gives higher vulnerability to organisms. In the same line, several classes, ways of administration, and infection models should be also studied, by inducing oral cavity, bloodstream, and vaginal, ocular, nails, and skin infections, once they are the most commonly effected tissues by these yeasts. Furthermore, the prophylactic effect of plant extracts (e.g., phenolic extracts) and related individual compounds should be also considered. In fact, the prevention of fungal infections might be as important as the therapeutic intervention.

Considering all the above described features, more detailed studies should be carried out, aiming not only at a better comprehension of the present problematic conditions, but also at providing new and more effective alternative treatments (including a prophylactic approach) and, lastly, at giving consistent data and specific tools towards a future approach regarding clinical trials.

## 4. Anti-*Candida* Species Inherent Mechanisms of Action

Although several studies have been carried out evaluating the antifungal mechanisms of action of natural matrices and isolated compounds, the experimental studies involving phenolic matrices with evidenced* in vivo* anti-*Candida* potential are considerably scarce. Among the* in vitro* determined anti-*Candida* potential of natural matrices, the effects on the immune system, namely, macrophage activation and upregulation of the expression of receptors related with phagocytosis, were one of the most accessed mechanisms [65]. Martino et al. (2011) concluded that the studied fractions of the aqueous extract of* Larrea divaricata* Cav. improve the superoxide anion production and, consequently, increase the phagocytosis of* C. albicans* and improved the nitric oxide (NO) production when compared with controls. Those fractions evidenced not only a direct action on macrophage activation, but also an indirect effect on production and releasing of ROS and NO, which enhanced the destruction of the invaders. On the other hand, Roy et al. [74], evaluating the antimicrobial and wound healing potential of* Pyrostegia venusta* Miers, proposed that the evidenced healing effects were related with the accumulation of the anti-inflammatory and proinflammatory cytokines and cells of immune system, particularly monocytes and macrophages, as also due to the antimicrobial effects of the tested natural matrix in the local of the tissue injury. In addition, Chen et al. [69], evaluating the wound healing and anti-inflammatory potential of* Lonicera japonica* Thunb. and related mechanism of wound contraction, concluded that these effects were mainly attributed to the improvement of collagen and granulation tissue formation, fibroblast proliferation, angiogenesis, and consequently stimulation of the reepithelialization. Furthermore, the authors proposed that these effects were also due to the existence of synergistic effects between antimicrobial and anti-inflammatory properties of the active ingredients, including chlorogenic acid.

Kumar et al. [67] evaluating the immunomodulatory potential of* Phyllostachys bambusoides* Siebold & Zucc, concluded that the observed effects were mainly due to the upregulation of the macrophage activation and, consequently, induction of the phagocytic activity. The observed effects were in part derived from the improvement of NO production. These facts resulted in a significant increase of the macrophage phagocytic activity of* Candida* species as well as in a promotion of the proliferation, maturation, and improvement of the immunological function of other immune defense cell types, resulting in an effective eradication of the invaders.

**Figure 2:**
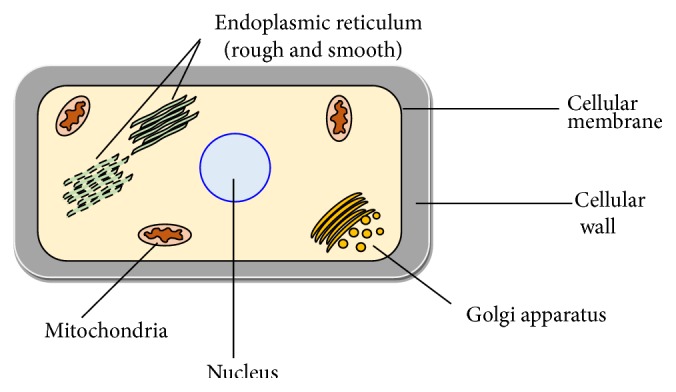
Mechanisms of action of the* in vivo* tested phenolic extracts and compounds against* Candida* species.

Otherwise, the effects of the phenolic extracts could be exerted directly in* Candida* species, instead of in the host individuals.  Figure 2 shows the most common sites of action of the antifungal drugs. The interaction with the fungal membrane and its specific components has been described as one of the most common targets of natural matrices. Associated with an increasing number of resistant-*Candida* species to the conventional antifungal agents, and reminding the predominance of critical infections, the discovery of new and effective alternatives is of the utmost relevance. It is a fact that several antifungal agents, such as azoles (e.g., fluconazole) and others that interact with fungal membrane, mainly exert fungistatic effects, despite their current higher rates of inefficiency. Among the reports about the anti-*Candida* activity of plant extracts, the associated modes of action of those plant extracts and even isolated compounds have been increasingly clear. For example, Jothy et al. [77] described a higher anti-*Candida* activity of the methanol extract of* Cassia fistula* Linn. seeds and carried out an evaluation of the mechanisms of action, by scanning electron microscope (SEM) and transmission electron microscope (TEM) observations. The authors verified that, in comparison with controls, treated cells presented a significant small size, irregular shape, with cell wall modifications, and clear depressions on the cell surface with holes. Furthermore, and after 12 h, the leakage of ions between treated and untreated cells appears to be the same; between 12 and 36 h, a significant increase in K^+^, Ca^2+^, and Mg^2+^ leakage has occurred in the treated cells. It means that, during the first 12 h, the applied extract exerts a little effect on the cell membranes but, after this time, its disruption associated with an important surface alteration and related damage were observed. As proposed by the authors, the anti-*Candida* activity of* C. fistula* may occur by two main modes: firstly, a passive entrance of the seeds extract into the plasma membrane, initiating the membrane disruption and, then, by the accumulation of the* C. fistula* seeds extract in the plasma membrane that results in cell growth inhibition.

Concerning to the mechanisms of action of isolated phenolic compounds, Li et al. [86], evaluating the activity of pterostilbene (PTE), a stilbene-derived phytoalexin, against* C. albicans* biofilms formation, observed a dose-dependent antibiofilm effect from the PTE concentration used. These achievements were mainly due to the disruption potential, induction of filamentous defects, and reduction of the cell density. In addition, the authors assessed the effect of PTE on gene expression and observed that, at 16 *μ*g/mL, 307 genes were differentially expressed: 193 were downregulated and 114 were upregulated. The observed downregulated genes were mainly related with the process of ergosterol biosynthesis, function of oxidoreductase activity, and components of the cell surface, while the upregulated genes were related with the process of protein unfolding (heat shock proteins).* RAS1* (the RAS signal transduction GTPase gene),* ECE1* (cell elongation protein gene),* SAP5* (secreted aspartyl proteinase gene),* SAM2* (S-adenosylmethionine synthetase gene),* PGA10* (involved in cell surface), and* ERG11* (involved in the ergosterol biosynthesis) are some of the downregulated genes. On the other hand, ESC4 (related with the inhibition of biofilm formation), YWP1 (encoding yeast cell wall protein), CEK1 and RIM8 (involved in filamentation), and HSP78 (involved in protein unfolding) were found among the upregulated genes. Furthermore, the authors described that the PTE also exerts an effect on the Ras/cAMP pathway, once after the PTE treatment and the exogenous cAMP restored the yeast-to-hypha morphological transition, and they also concluded that PTE was able not only to inhibit the biofilm formation but also to destroy the maintenance of mature biofilms. Lastly, by studying the* in vivo* antibiofilm potential of PTE, the authors confirmed all of the above proposed theories and also observed that PTE presents a high bioavailability and no toxicity on the tested mice models [86].

In the same line, Sharma et al. [88], studying the* in vivo* antifungal potential of curcumin and its mode of action, concluded that curcumin acts by mediating the ROS signaling pathway and, consequently, stimulates the proapoptotic regulatory processes by increasing the number of preapoptotic cells. Notwithstanding, the authors also described that the addition of an antioxidant could prevent these effects. Furthermore, it was showed that curcumin modulated the drug efflux of yeast ABC transporters without affecting the levels of transcription genes encoding these transporters. A block of the hyphae growth was also reported by the authors, not only in* C. albicans* but also in other non-*albicans Candida *species, and in this case, the addition of antioxidants could not reverse the inhibitory effect. Lastly, the authors verified that curcumin targeted the global repressor TUP1 (thymidine uptake 1) which, in an independent manner of ROS production, prevented the hyphae development, in both liquid and solid hypha-inducing media [88].

Taking into account the above-described mechanisms of action of phenolic extracts and related individual compounds, it is feasible to conceive that plant extracts could be promisor antifungal agents. Moreover, no toxicity was found to the majority of the tested phenolic matrices, which itself opens new perspectives for the future approach as new anti-*Candida* leaders.

## 5. Current Phytochemical Formulations

Several phytochemical preparations are currently available, for either external or internal uses [89]. Creams, lotions, powders, sprays, and ointments are mainly directed for cutaneous mycosis and onychomycosis, while suspensions, capsules, drops, or suppositories have been used for mucous infections.

Table 3 shows the most frequent* in vivo* phytochemical preparations for intraperitoneal, intravaginal, intravenous, oral, and topical uses. Oral administration, followed by topical, intraperitoneal, intravaginal, and intravenous administrations, is one of the most common administration ways. For oral administration, several types of suspensions, aqueous solutions, direct-plant extract application, and mouthwashes were also tested. In relation to mouthwashes, toothbrushes with toothpaste and interdental cleaners are, currently, the most common procedures in order to maintain a correct oral hygiene. However, and despite some mouthwashes and mouthrinses being also available, alcohol is commonly used in order to ensure a correct dissolution of the active ingredients. Its use has been questioned because the alcohol-containing mouthwashes induce desiccation of the oral mucosal membranes. Thus, it is very important to prevent, reduce, and even treat plaque microbial-associated diseases but also to ensure that the used preparations are naturally safe and efficient. In this sense, a mouthwash solution was applied by Chelli-Chentouf et al. [81] in a children's school, in order to evaluate not only its efficiency but also its stability and physicochemical parameters.

Creams and ointments are the most studied formulations for topical use in fungal infections, mainly in cases of local affections, such as excision and incision wounds, and vaginal infection models. In the last case, intravaginal administration is the highly indicated, as also cream, solution, or even suspension [70, 79]. Nevertheless, other phytochemical preparations have also been administered in laboratorial models, namely, by intraperitoneal [65, 75, 77] and intravenous [78] ways.

It is convenient to highlight that the laboratorial formulations are specifically designed according to the examined clinical models; that is, for local/topical uses, creams, ointments, and also intravaginal administration comprise the first choice to evaluate the wound healing and antifungal potential in externally affected models, while suspensions, solutions, and mouthwashes are specifically indicated for internal uses (oral, intraperitoneal, and intravenous) in laboratorial models, such as in cases of induced systemic infections, immunomodulation, anti-inflammatory, and antinociceptive, and in some cases healing potential. In addition, in some phytochemical preparations, histological and histopathological studies were also carried out [68–75]. The evaluation of the tissues architecture between controls, pretreated and treated models, is of extreme relevance. The obtained results by these studies allow an effective determination of the toxic potential but also predict its possible types/ways of use, providing upcoming information related with its possible clinical applications.

## 6. Conclusion and Future Perspectives

Microorganisms are able to provide several benefits to the host (due to the establishment of symbiotic relationships) but are also responsible for severe health conditions. Antimicrobial agents are normally effective, but due to their overuse and related side effects and toxicity, its effectiveness has been seriously questioned. Plants phenolic extracts and isolated compounds possess a multitude of healing properties. Methanol, followed by acetone, water, ethanol, petroleum ether, methanol-dichloromethane, ethyl acetate, and n-butanol extracts obtained from leaves, seeds, fruits, and flowers comprise extremely enriched-phenolic sources. Despite the pool of phenolic compounds present in phytochemical preparations, only stilbenes (namely, pterostilbenes and riccardin D) and curcuminoids (curcumin and piperine) have been studied for their* in vivo* anti-*Candida* effects. Oral (suspension, solution, mouthwash, and crude extracts), followed by topical (ointment and cream), intraperitoneal (solution and suspension), intravaginal (cream, solution, and suspension), and intravenous (solution), phytochemical formulations were often prepared for* in vivo* administration.

In fact, phytochemicals are the basis for the design and development of new synthetic drugs, but while synthetic chemical drugs act by a single way, natural matrices are able to establish synergisms, antagonisms, and even polyvalence effects*. In vitro* studies are normally the first choice to evaluate the healing properties of natural matrices. However,* in vivo* studies present a higher importance, once considers the organic metabolism and pharmacokinetic and pharmacodynamic parameters, among other factors.

Overall, and apart from the stated advances, upcoming specific clinical formulations, ways of administration, and therapeutic dosage need to be established, being clinical trials very important to deepen knowledge through more detailed* in vivo* studies.

## Figures and Tables

**Table 1:** Phenolic extracts with *in vivo* activity against *Candida* species.

Family	Species	Part	Preparation	Condition	*Candida* spp.	Animal model	Exposure	Doses	Bioactive	Ref.
Acanthaceae	*Justicia flava *(Forssk.) Vahl	Leaves	Methanol extract	Wound healing	*C. albicans*	Sprague-Dawley rats	24 hours, 24 days	7.5% w/w extract aqueous cream	Alkaloids, flavonoids, steroids, carbohydrates, sapogenetic glycosides, and tannins	[73]

Anacardiaceae	*Lannea welwitschii *(Hiern) Engl.	Leaves	Methanol extract	Wound healing	*C. albicans*	Sprague-Dawley rats	24 hours, 24 days	7.5% w/w extract aqueous cream	Alkaloids, flavonoids, steroids, carbohydrates, sapogenetic glycosides, and tannins	[73]

Arecaceae	*Elaeis guineensis *Jacq	Leaves	Methanol extract	Antifungal potential	*C. albicans*	Swiss albino mice	Once daily during 7 days	Oral administration: 2.5 g/kg body weight	Phenols	[76]

Bignoniaceae	*Pyrostegia venusta *Miers	Flowers	Methanol extract	Antimicrobial and wound healing potential	*C. albicans *and* C. tropicana*	Wistar rats	19 days	100 mg/kg body weight	n.d.	[74]

Caprifoliaceae	*Lonicera * *japonica* Thunb.	Flowering aerial parts	Ethanol extract	Antiwound infection, repair, and contraction	*Candida albicans, C. tropicalis*	Wistar rats	15 days	10% (w/w) LJEE ointment	Chlorogenic acid	[69]

Combretaceae	*Combretum albopunctatum *Suesseng *Combretum imberbe *Wawra *Combretum nelsonii *Duemmer *Terminalia sericea *Burch ex DC	Leaves	Acetone extract	Wound healing and antifungal potential	*C. albicans*	Immunosuppressed Wistar rats	3 times a week, during 17 days	20% in aqueous cream	n.d.	[71]

Eriocaulaceae	*Syngonanthus nitens *(Bong.) Ruhl.	Scapes	Methanol extract	Vulvovaginal candidiasis	*C. albicans*	Wistar rats	2 times per day, 7 days	Cream with 0.5%, 1.0%, and 2.0% of extract	Flavonoids, flavone derivatives	[70]

Leguminosae	*Cassia fistula *Linn.	Seeds	Methanol extract	Anticandidal potential	*C. albicans*	Swiss albino mice	Once daily, during 3 days	2.5 g/kg body weight (intraperitoneal)	n.d.	[77]

Meliaceae	*Swietenia mahogani *(Linn.) Jacq.	Seeds	Methanol extract	Antifungal potential	*C. albicans*	Mice	7 days	2.5 g/kg of extract (intraperitoneal)	Saponins, phenols, volatile oils, alkaloids, anthraquinones, and terpenoids	[75]

Moraceae	*Ficus glomerata *Roxb.	Fruit and bark	Methanol extract	Immunomodulatory potential	*C. albicans*	Swiss albino mice	13 days	250 and 500 mg/kg p.o	Carbohydrates, glycosides, wax, steroids, saponins, and tannins	[66]

Olacaceae	*Olax subscorpioidea *var.* subscorpioidea *Oliv.	Fruits	Methanol-dichloromethane (3 : 1 v/v) extract	Anti-*Candida* potential (systemic candidiasis)	*C. albicans*	Female albino Wistar rats	3 days	0.5, 1 and 2 g/kg of body weight, i.v. administration	n.d.	[78]

Parmeliaceae	*Pseudevernia furfuracea *(L.) Zopf	Lichens (thallus)	Methanol, dichloromethane, ethyl acetate, and n-butanol extracts	Anti-inflammatory, antinociceptive, and wound healing potential	*C. albicans, C. dubliniensis, *and* C. krusei*	Sprague-Dawley rats and Swiss albino mice	Between 60 min and 9 days, according to the studied bioactivity^1^	Oral administration and topical use	Isolated compounds: atraric acid, mixture of methyl hematommate and methyl chlorohematommate	[72]

Poaceae	*Phyllostachys bambusoides *Siebold & Zucc.	Leaves	Ethyl acetate fraction of the alcoholic extract	Immunomodulatory potential	*C. albicans*	BALB/C mice	5 days	Oral administration: 50–200 mg/Kg body weight	Isoorientin, orientin, and isovitexin	[67]

Polygonaceae	*Persicaria senegalensis *(Meisn.) Soják	Leaves	Cooked leaves Leaf powder	Anti-*Candida* activity (subclinical mastitis)	*C. albicans*	Lactating cows of three farms	5 days	1.5 kg/day 0.77 kg/day	n.d.	[80]

Punicaceae	*Punica granatum *L.	Peels	Aqueous: methanol (75%) extract	Wound healing potential	*C. albicans *and* C. glabrata*	Guinea pigs	Once daily during 10 days	5% (w/w) methanolic extract based ointment, using soft white paraffin, as vehicle	Ellagitannins (punicalagin A, punicalagin B, gallic acid, and ellagic acid), followed by anthocyanidins	[68]

Salvadoraceae	*Salvadora persica* L.	Chewing sticks	Methanol extract	Oral health of children presenting decays	*C. albicans *and* Candida *spp.	School children aged between 6 and 12 years	1 week	Mouthwash	Phenolic compounds	[81]

Verbanaceae	*Clerodendron splendens *G. Don	Plant material	Methanolic extract	Wound healing and antifungal effects	*C. albicans*	Sprague-Dawley rats	9 days	100 mg of ointment (33.3% w/w *C. splendens* extract in simple ointment BP), twice daily	Reducing sugars, phytosterols, tannins, terpenoids, alkaloids, and flavonoids	[90]

Vitaceae	*Vitis vinifera *L.	Seeds	Ethanol : water (7 : 3 v/v) extract	Antifungal potential (vaginal candidiasis model)	*C. albicans*	CD1 mice	Every two days, until day +8	10 *µ*L/mouse (50 mg/mL) intravaginal	Mixture of monomeric, oligomeric, and polymeric flavan-3-ols	[79]

Zygophyllaceae	*Larrea divaricata *Cav.	Leaves and tender branches	3 fractions of the aqueous extract	Immunomodulatory potential (innate immunity enhancer)	*C. albicans*	Rockland mice	twice in a 48-hour period	0.5, 5 and 15 mg kg^−1^ of fractions, intraperitoneal	n.d.	[65]

^1^Anti-inflammatory: every 90 min during 6 h after induced inflammation, antinociceptive: 60 min after oral administration of test samples, counted for the following 15 min (starting 5 min after the PBQ injection), wound healing activity: once a day, during 9 days, and n.d.: not determined/detailed.

**Table 2:** Phenolic compounds with *in vivo* activity against *Candida* species.

Compound	Class	Origin	Condition	*Candida* spp.	Purpose	Animal model	Exposure	Doses	Reference
Curcuminoids: curcumin and piperine	Polyphenols	Commercial standards	Systemic murine model of infection	*C. albicans*	Antifungal potential and related modes of action	Swiss albino mice	1st dose + 2nd dose after 6 h of the first, during 2 days	Curcumin alone: 100 mg/kg of body weight Curcumin + Piperine: 100 and 20 mg/kg of body weight	[88]

Pterostilbene (PTE)	Stilbene-derived Phytoalexin	Laboratorial synthesis	Central venous catheter infection	*C. albicans*	Antibiofilm potential	Sprague-Dawley rats	72 h of incubation	500 *µ*L liquid containing different concentrations of PTE (0, 16, 32, and 64 *µ*g/mL)	[86]

Riccardin D (RCD)	Macrocyclic bisbibenzyl	Chinese liverwort *Dumortierahirsute*	Intravenous catheter infection	*C. albicans*	Antibiofilm (prophylactic and therapeutic) potential	New Zealand white rabbits	Prophylactic: 8 h Therapeutic: 8 h per day during consecutive 5 days	Prophylactic: Groups II, III, and IV, 300 *µ*L of RCD solution (8, 16, and 64 *µ*g/mL, resp.), Group VI, 300 *µ*L of FLC (4 *µ*g/mL) plus RCD solution (16 *µ*g/mL) Therapeutic: 300 *µ*g (8, 16, and 64 *µ*g/mL, resp.) was injected after 24 hours of infection	[87]

**Table 3:** Phytochemical preparations evaluated through *in vivo* laboratorial models.

Application	*In vivo* model	Type of formulation	Reference
Intraperitoneal	Immunomodulation	Solution (water)	[65]
	Intravenous *Candida* infection	Solution (methanol)	[77]
		Suspension (methanol)	[75]

Intravaginal	Vaginal infection	Cream (o/w emulsion)	[70]
		Suspension	[79]

Intravenous	Intravenous *Candida* infection	Solution (water)	[78]

Orally	Anti-inflammation, antinociceptive and would healing potential (incision/excision models)	Suspension (0.5% sodium carboxy methyl cellulose (CMC))	[72]
	Excision and incision wound	Suspension (extract in vehicle)	[74]
	Immunomodulation	Suspension (1% sodium carboxy methyl cellulose (CMC))	[66]
		Suspension (1% w/v aqueous gum acacia)	[67]
	Intravenous *Candida* infection	Solution (aqueous)	[76]
	Oral cavity children affections	Mouthwash (water)	[81]
	Subclinical mastitis	Crude extracts (cooked and powder leaves)	[80]

Topically	Excision and incision wound	Ointment (vehicle)	[72]
		Cream (aqueous)	[71]
	Excision wound	Cream (aqueous)	[73]
		Ointment (w/w)	[69]
		Ointment (w/w)	[90]
		Ointment (vehicle)	[68]
